# Digital ethical reflection in long-term care: Leaders’ expectations

**DOI:** 10.1177/09697330231191276

**Published:** 2023-08-04

**Authors:** Lena Jakobsen, Rose Mari Olsen, Berit Støre Brinchmann, Siri Andreassen Devik

**Affiliations:** 1786 Nord University, Norway; Centre for Care Research Mid-Norway, 158928 Nord University, Norway; 1786 Nord University, Norway and Nordland Regional Hospital, Norway; Centre for Care Research Mid-Norway, 1786 Nord University, Norway

**Keywords:** Ethical reflection, digital tool, leadership, home care

## Abstract

**Background:**

Healthcare leader support and facilitation for ethics work are of great importance for healthcare professionals’ handling of ethical issues, moral distress, and quality care provision. A digital tool for ethical reflection in long-term care was developed in response to the demand for appropriate tools.

**Research aim:**

This study aimed to explore healthcare leaders’ expectations of using a digital tool for ethical reflection among their home nursing care staff.

**Research design:**

A qualitative research design with vignettes and focus group interviews was used. The data were analyzed using reflexive thematic analysis.

**Participants and Research Context:**

The sample comprised ten healthcare leaders from municipal long-term care in Norway.

**Ethical Considerations:**

Participation was voluntary and based on informed consent. The Norwegian Agency for Shared Services in Education and Research approved this study.

**Findings:**

The analysis resulted in the development of three themes representing leaders’ expectations of the tool: (i) giving staff a voice, (ii) revealing ethics in practice, and (iii) keeping the pot boiling.

**Discussion:**

Leaders must be informed about the ethical issues experienced to engage in supportive measures for their staff. Digital channels can increase the democratization, safety, and efficiency of communication between employees and leaders and increase awareness of ethical aspects in daily care work. The leaders had positive expectations of employees’ activities and benefits from using the tool for ethical reflection. However, they admitted that realizing the tool’s potential to ensure continuity in ethics work requires considerable organizational and leader involvement.

**Conclusions:**

This study shows that healthcare leaders are open to digital solutions for managing ethics work in home nursing care. Both opportunities and important prerequisites for successfully implementing the digital tool were identified.

## Introduction

The need for ethical support for healthcare professionals is well described in the literature; however, no single method or approach has proven to be the best for reducing or helping healthcare professionals handle moral distress.^1^ The degree of support offered by an organization and its leaders impacts how healthcare professionals experience moral stress.^2^ Therefore, Jakobsen et al. developed a digital ethical reflection tool to promote nurse leaders’ work with ethics support among healthcare professionals in municipal long-term care settings.^3^ The digital ethical reflection tool is an online survey containing questions about ethical issues healthcare professionals encounter during their shifts. Its responses then constitute a basis that leaders can use to improve ethics work at the work unit level (e.g. through ethical group reflection). In a previous article, Jakobsen et al. explored and described the functionality and utility of the digital ethical reflection tool from the perspective of healthcare professionals. This article adds new knowledge to the field of ethics work in municipal long-term care by exploring nurse leaders’ views and expectations regarding implementing the digital ethical reflection tool in home nursing care. While a previous article by Jakobsen et al. ^3^ examined the functionality and utility of the digital ethical reflection tool from the perspective of healthcare professionals, this study explores the views and expectations of nurse leaders regarding its implementation in home nursing care. By understanding nurse leaders’ perspectives, this research contributes to the development of effective strategies for implementing and optimizing the digital ethical reflection tool. The findings of this study have the potential to enhance ethics support for healthcare professionals and improve the overall quality of nursing care in municipal care settings.

## Background

Ethics support for healthcare professionals in municipal long-term care is characterized by fragmented and unstructured approaches,^4^ leaving them feeling unsafe and faced with difficulties complying in a caring nursing environment.^5,6^ Ethical competency is vital for quality nursing care,^7^ and the ability to make ethical decisions in practice is an important skill for healthcare professionals. In this context, a nurse leader plays an important role. There are many different approaches to ethics support in healthcare. The most frequently used approaches include clinical ethics committees, ethics consultants, and ethical reflection groups.^8^ An increasingly ageing population and the need for in-home advanced treatment contribute to ethical issues surrounding municipal long-term care.^9^ In home nursing care, nurses are expected to deliver quality care during time restrictions^10^ and fulfil patient needs alone in the patient’s home.^11^ The strain of this can sometimes feel overwhelming and challenging for nurses.^12,13^ However, reflecting upon these experiences can help increase moral awareness and understanding of patients and colleagues.^14^

Moral distress, as a complex psychological response, has been recognized as a prevalent issue in the nursing field. According to Morley,^15^ moral distress refers to the psychological stress experienced as a consequence of encountering moral situations characterized by moral tension, moral conflict, moral dilemma, moral uncertainty, or moral limitation. This distress can manifest in various negative emotions, including anger, frustration, guilt, sadness, and a sense of powerlessness.^15^ Over time, nurses may internalize these constrains, thereby further contributing to their experience of moral distress.^16^ Addressing moral distress among healthcare professionals should be a priority for organizations, motivating them to provide recourses to assist staff in dealing with this distress.^1,17^ Additionally, experiences of moral distress can also serve as opportunities for professional development and self-improvement.^18^ Enhancing nursing leaders’ ability to work with programmes or interventions that improve staff wellbeing is a sustainable approach for retaining and recruiting healthcare professionals.^19^ Furthermore, this approach empowers nurse leaders themselves.^20,21^ Prior research has typically focused on how ethical leadership contributes to the organization’s psychological, personal, and ethical climate, thereby influencing patient health outcomes and the quality of nursing care.^22,23^

Nurse leaders have the opportunity to help healthcare professionals build moral resilience.^2^ Assisting healthcare professionals in developing competence in dealing with ethical issues is an important aspect of a leader’s role.^4^ Studies have shown that nurse leaders are important role models in facilitating support for ethical decision making among staff,^24,25^ being clinically responsible for the care in the work unit,^26,27^ and providing an ethical work climate.^28^ Both nurse and organizational leaders must acknowledge their responsibility in providing ethical support to staff.^4,29–31^

Long-term care provides a diverse array of personal, social, and medical services and supports individuals with physical or mental limitations to maintain their function and dignity.^32^ In Norway, the municipalities are responsible for organizing and providing long-term care for their inhabitants, a service which has expanded in the last decade.^33^ Long-term care is typically organized in geographic sectors in which nurses have a base where they meet between patient home visits. The nurse leader’s office is located at this base.^10,34^

Being present and approachable in the work environment make staff feels closer to the nurse leader. In 2013, Storch et al.^28^ reported how nurses feel a lack of ethical support from their leaders, despite it being the nurse leaders’ responsibility to provide this support. In 2020, Devik et al.^4^ stated that this problem persisted among nurse leaders, who struggled to find a strategy for executing ethical leadership. The diverse demands on nurse leaders include maintaining economic efficiency and attending to patient and staff needs.^35^ Considering the nurse leaders’ responsibilities and expectations from both the organization and staff, studies need to investigate novel solutions to support their ethics work. The digital ethical reflection could offer several opportunities to enable this support and need to be examined. Therefore, this study aimed to explore the expectations of nurse leaders in home nursing care about using digital ethical reflection for ethics work. Its research question was, ‘how can digital ethical reflection act as an ethics support tool for home nursing care leaders?’.

### The digital ethical reflection

In recent years, digital solutions have been investigated, primarily in the contexts of enhancing ethical competence among nursing students^36^ and ethical decision-making capabilities among nurse leaders.^37^ Digitalization has been positively embraced within the nurse education system in particular, partly due to the COVID-19 pandemic,^38^ and by nurses involved in patient healthcare.^39^

**Figure 1.**
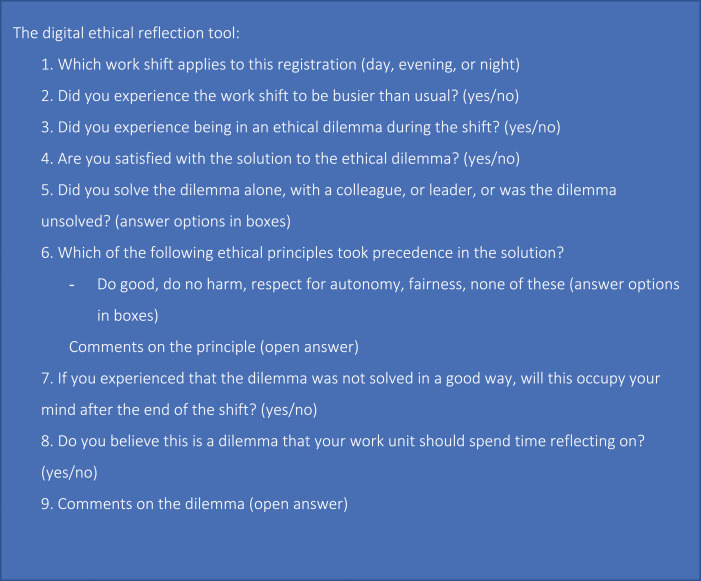
Questions in the digital ethical reflection tool.

The digital ethical reflection tool is an anonymous online digital tool for registering and describing the ethical challenges and dilemmas experienced by nurses during a shift (Figure 1). The tool was field tested by 17 nurses working in nursing homes and home nursing care in Norway.^3^ The tool was developed to serve multiple purposes. It should serve as an outlet for moral distress among staff, stimulate employees’ moral sensitivity, and give leaders insight into staff`s experiences as a starting point for targeting ethical follow-up work, such as ethical group reflection. The digital ethical reflection tool comprises nine questions and is based on the four biomedical ethical principles described by Beauchamp and Childress.^40^ These principles were chosen because they are familiar and understandable to personnel in this context and would allow us to categorize the responses.

We are well aware that these principles originate from the medical field and have been criticized for not fully covering the everyday ethics nursing staff may experience.^41^ At the same time, we needed to create standardized questions that could give us (and the leaders) insight into what the challenges were about in an efficient way (i.e. in digital format). The digital format also required simplicity and that the participants could efficiently carry out the registration. Opportunities for participants to answer in their own words were also provided. Questions and answer options were found relevant among the health workers who had previously tested the tool in the pilot. The data provided by the healthcare professionals is intended to be summarized by the project leader and reported back to the nurse leader monthly. The answers provided are securely encrypted and stored in ‘Nettskjema’ at the University of Oslo. The tool is not considered an actual reflection. Rather, it is to be used by the nurse leader as a starting point for ethical group reflection at the work unit.

## Methods

### Research design

This was a descriptive qualitative study in which the phenomenon of interest was described using everyday language.^42^ The participants’ experiences and views were obtained through focus group interviews to answer the research question. We chose focus group interviews since the group process and participant interactions could provide additional information on the research question.^43^ Appreciating that participants’ thoughts, feelings, and experiences are unique, we adopted a critical orientation to ensure the discourse was constructive. In addition, as researchers, we approached the reality constructed by the participants.^44^

### Study setting, sample, and method of data collection

Participants were selected using a purposive sampling method, which is considered a valid approach since it might increase the available information.^45^ Invitations to participate were sent to the department heads of home care services across six municipalities in Norway. The municipalities were recommended by an ethics consultant from The Norwegian Association of Local and Regional Authorities (KS). In Norway, nursing homes and home nursing care organizations are the public responsibility of each municipality. KS is the organization for local governments in Norway, and all municipalities and county councils are members. One male and nine female leaders from two municipalities representing fourteen work units consented to participate (Table 1). The participants were responsible for 14–65 employees in their respective work units and had worked as leaders for 0.5–8 years. Nine leaders were undergoing continued education, and seven had received specific leader education. Sample homogeneity was ensured by all participants being from home nursing care work units and registered nurses or social educators.^1^

**Table 1.** Overview of the focus group participants.

Focus groups	General home nursing care (n)	Substance abuse and mental health care (n)	Meeting place	Total participants
Group 1	2		Town hall meeting room	2
Group 2	2	1	Town hall meeting room	3
Group 3	3	1	Digital	4
Individual interview		1	Town hall meeting room	1

### Focus group interviews with vignette

The focus group interviews took place in May 2022 in neutral town hall meeting rooms in each municipality. Some participants were familiar with each other through work connections; however, most did not have any prior connections with other participants besides having potentially met during work meetings, courses, and other events. The interviews were semi-structured and led by LJ as the moderator and SAD as the co-moderator.

**Figure 2.**
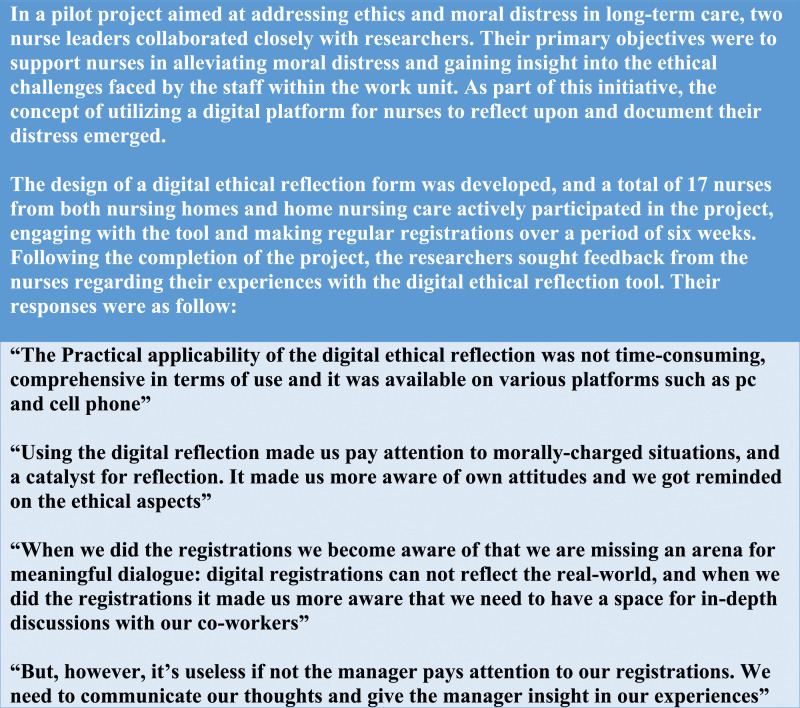
Vignette presented to the participants.

We initially presented experiences from nurses who had tested the digital ethical reflection tool^3^ as a vignette, demonstrating how important nurse leader involvement is for the ethical support of nurses (Figure 2). Using a qualitative vignette approach in the focus group interviews enabled deeper contemplation from the participants on the presented topic. The vignette method can capture participants’ meanings when they respond to a presented short story.^46^

After introducing the vignette, the participants were asked, ‘which possibilities could digital ethical reflection have for you and your work unit?’ with follow-up questions such as ‘could you deliberate that further?’. The interactions between participants appeared positive; all participants contributed, and while the conversation strayed at times, it largely was kept within the topic. All four interviews lasted 30–75 min and were recorded using the ‘Nettskjema-dictaphone’ app. Recordings were encrypted and stored at the University of Oslo before verbatim transcription.

### Data analysis and interpretation process

By using the reflexive thematic analysis (TA) approach described by Braun and Clarke,^44^ we acknowledged the researcher’s active role in knowledge production. The orientation to TA when processing and producing research data outcomes is an important part of reflexive TA.^47^ Our background as registered nurses with work experience in home nursing care and management experience further reflected our position and understanding of the data. The point of departure for the analysis was how digital ethical reflection can act as an ethics support tool for home nursing care leaders.

Data interpretation followed the six-step approach described by Braun and Clark,^44^ beginning with familiarizing ourselves with the data before identifying patterns of meaning in the text and coding the features. The codes were then connected into potential themes. Following this, all authors reviewed, defined, and refined the themes, after which LJ wrote the report. At this final step, the research question was revisited, and the whole story was examined for consistency by all researchers, increasing the credibility of the findings. The analysis fluctuated between manifest and latent meaning coding throughout the process.

### Ethical considerations

All participants were informed about the withdrawal possibilities, voluntary participation nature, and confidentiality rules. Written informed consent was obtained from all participants before the interviews.

## Findings

Three main themes were developed: giving the staff a voice, revealing ethics in practice, and keeping the pot boiling.

### Giving the staff a voice

The participants were concerned with ensuring that all the staff members were heard. Letting the staff have a say on how they perceive their work was considered part of the leadership role. Several participants found having good conversations with some staff challenging due to their secluded nature in staff meetings and one-on-one annual supervisor conversations. According to some participants, no matter how strong and confident some staff members are when speaking, some always shut down or do not have enough confidence to say how they feel.

‘For those who find it a bit difficult…we are different persons, and some think it’s difficult to speak up during the lunch meetings and to even say something at all. Maybe it’s easier for them to process things when it becomes digital, and you don’t have to speak up in a big congregation. I believe my staff will find that useful’ (Group 1).

Most participants felt they had a responsibility to be informed and aware of what happens within their work unit, the challenges, and how the ‘temperature’ was among staff. If a leader has insight into what is going on, there is an opportunity to ‘catch’ any potential conflict involving ethical issues before it escalates and negatively impacts the whole work unit.

‘I think my staff is very occupied by being nice to each other, no matter what. I mean, of course, we are nice even if we tell our leader about problems … but I have been told way too late when they have experienced an ethical dilemma. If I’d known earlier on, I could have done something about it, ‘cause then they have been stuck in it for a long time and are worn out’ (Group 2).

Sometimes, ethical challenges were put aside because staff focused on getting home after their shift.

‘I often have staff coming to me many days later telling me that at this shift, this and that happened…It’s like when things actually happen it kind of just gets ignored, and they just want to get home after their shift is finished. It’s when they get landed afterwards; they start to feel that this was demanding or need to bring it back up’ (Group 3).

The participants highlight the significance of giving the staff members a voice and ensuring active participation in expressing their perspectives. Additionally, the participants also emphasize the importance of leaders being informed about the dynamics within their work units to address ethical challenges proactively.

### Revealing ethics in practice

Most participants stated that their rationale for participating in the digital ethical reflection project was increasing ethical awareness and improving decision-making skills among their staff. One of the leaders stated, ‘I think that the goal of doing this is for all of us to be good at recognizing an ethical dilemma and try to resolve it’. Furthermore, some leaders suggested that using the tool in the work unit could help staff become more confident in making good choices when encountering ethical challenges.

Many participants felt that digital ethical reflection could help staff become more aware of and learn to identify ethical issues. Learning to recognize ethical issues was mentioned by most participants as a key reason for introducing digital ethical reflection.

‘I am eager to start the work again and keep attention to ethics! ‘Cause it’s difficult sometimes to distinguish between what’s an ethical challenge and what’s just a practical issue from everyday work practice’ (Group 1).

Some participants also voiced that they felt there was a level of unawareness among their staff regarding ethical values. Many participants were concerned that their staff did not use value-based language and spoke unfairly of other people and patients. Leaders hoped that using digital ethical reflection would improve inadequate ethical language use.

‘I know there will be staff with poor language comprehension who will struggle in the beginning with understanding the ethical principles in the digital reflection. I think I will start by explaining and actually use the ethical principles in everyday speech, at least in my work unit’ (Group 3).

According to most participants, even staff with adequate ethical language comprehension struggled to understand the concept of ethical dilemmas and separate ethical reflections from everyday conversations. The participants expressed concerns regarding the staff’s ability to recognize ethical aspects and dilemmas in their daily practice.

‘I don’t believe that when my staff bring up in the reports something they want to discuss, they necessarily consider whether it’s ethical or not. It’s more about their thoughts, struggles, and concerns- something they feel stuck in’ (Group 2).

The participants believed that when introducing digital ethical reflection, the digital format would spark curiosity and interest among the staff, leading to increased ethical awareness, improved decision-making skills, and enhanced recognition of ethical issues among the staff. The leaders expressed expectations about ethical development in staff both as a result of the tool itself and the discussions the tool would catalyze.

### Keeping the pot boiling

Most participants expected digital ethical reflection to help maintain a focus on ethics for staff and themselves. Many participants expected that working with feedback from digital reflection would help them systematize and schedule ethics work within their work units.

‘Then, when they use the digital reflection, it will be easy to see when things change because it will be visible to me. One month the digital ethical reflection’s statistics show its autonomy, which is reported most, but next month is something else. So, what’s happened here? Is it a new patient? Why have things changed?’ (Group 1).

The participants wanted staff to complete digital ethical reflection when their shift ended to deal with any issues and ensure they were informed of these issues in their capacity as a leader, so they could be prepared and know what needed to be discussed. While some participants had a clear plan for using digital registrations in follow-up ethical group reflections, others were more unsure about how they could or should practically use the information. Most participants believed that information registration had to become a procedure in which registrations occur in certain weeks and at regular intervals. Creating a system would provide predictability and ensure that registrations were not forgotten.

Some participants were worried about motivating staff to participate in digital ethical reflection. Several participants suggested that allying with a staff member could provide further support for implementing the tool. While many participants had a professional development nurse who helped maintain focus and conduct professional development in the work unit, others brought in external ethics consultants when they faced complex ethical issues. Participants who lacked a dedicated resource to confer with or the opportunity to engage external ethics consultants said they would discuss the tool with a staff member they trusted.

‘I am thinking about confiding and teaming up with one of the staff who is from another country and works night shifts. He has another way of approaching things than I do, and I think it would be exciting to see what he thinks’ (Group 3).

Some participants felt that keeping staff motivated was more manageable if they shared the responsibility of introducing, implementing, and executing the digital reflection with someone else. However, some participants expected healthcare professionals to take some personal responsibility as well and demonstrate an interest in improving their ethical practice.

‘We do some ethical reflections when we meet during the changes of shift, and I have really tried to get started for real. I experience it to be some contradictive oppositions among the staff … they say they want to have ethical reflection, but when I facilitate time for it, they don’t seem to care’ (Group 3).

Some participants thought using a novel method, such as digital reflection, to engage healthcare professionals would help spark and develop an interest in completing ethical reflection. Several participants thought that by making staff register their ethical dilemma experiences, they would learn to take personal responsibility for the reflection. Some participants thought this would improve staff accountability since completing their registrations would be expected.

‘I think this means they have to do some reflections on their own, not just air their thoughts of everything all the time…I think it’s useful for them to have to do the digital registrations; it means they have to focus on how they think by themselves’ (Group 2).

Many participants believed that incorporating personal responsibility through digital registrations would enhance staff engagement and promote individual reflection.

The participants expected the introduction of digital ethical reflection to help maintain focus on ethics. They believed working with the feedback from the tool would facilitate systematization and scheduling of ethics-related activities. Motivating staff to engage required strategic efforts, and building alliances was considered valuable to facilitate the implementation process.

## Discussion

The findings revealed three key themes that reflected the leaders’ expectations of the digital tool: giving the staff a voice, revealing ethics in practice, and maintaining a focus on ethics. These findings highlight the importance of empowering staff, enhancing ethical awareness, and promoting staff engagement in ethical reflection, thus supporting the expectations towards digital ethical reflection as a valuable tool for home nursing care leaders.

The leaders acknowledged a lack of opportunities and space for healthcare professionals to have their voices heard and express ethical concerns. Interpersonal dynamics among healthcare professionals can negatively affect their motivation to voice concerns.^48^ Healthcare professionals can find it difficult to speak up due to workplace hierarchies and their cultural backgrounds. Those with higher education levels and seniority are more likely to voice concerns and opinions.^49^ When healthcare professionals refrain from addressing ethical issues, such as unsafe practice by colleagues, or experience ethical challenges, it potentially threatens patient safety ^48^ and can increase their moral distress.

Facilitating a channel (e.g. digital ethical reflection) that could enable leadership support can help healthcare professionals feel more connected to the organization and foster an open culture within the work unit while maintaining individual anonymity for registrations in the digital ethical reflection tool. Using digital reflection to foster staff connections with their leader can increase communication safety and effectiveness. Leaders are faster in taking action if they get a report of misconduct or ethical misbehaviour than the healthcare professionals who encounter the problem.^50^ For leaders to support healthcare professionals, they must be informed of the ethical issue. Leaders who show interest in their staff’s wellbeing and problems and who manage to integrate the core values of the nursing profession among healthcare professionals act as good ethical role models and can influence healthcare professionals’ involvement in patient care.^29^

Storaker et al.^51^ described how nurse leaders and nurses struggle to identify everyday ethics in somatic hospital wards. While there is scant research on ethics management among healthcare professionals in community settings, in 2019, Porr et al.^52^ presented an explanatory theoretical model called Moral Compassing. This model explains how nurses ‘mull things over in their mind as they carry out an internal dialogue’ as a reaction when something feels wrong before seeking validation and mobilizing support for action or inaction. This self-talk helps healthcare professionals sort and differentiate the ethical issue. The leaders in our study expected their staff to initiate self-talk to subsequently separate ethical issues and practical problems when registering their experiences in the digital ethical reflection tools.

The utilization of the digital tool for identifying challenges and engaging in ‘self-talk’ to discern the underlying values necessitates validation and the mobilization of support within the working community, such as the establishment of an ethical reflection group. However, it is important to note that the discovery and discussion of ethical issues may not always align with the prevailing cultural norms in the workplace or the organization.^52^ Hence, leaders' recognition, facilitation, and active participation in ethical initiatives assume critical significance for fostering a culture of ethical reflection in the workplace and cultivating the moral compass of the staff. In this regard, the digital tool can serve as an innovative aid, particularly during the initial stages as described by Porr et al.^52^ as the process of ‘undergoing a visceral reaction’ and ‘self-talk’.

How individuals address each other reflects their values. Rather than using ethical concepts, leaders prefer to use common and value-based language to make invisible attitudes visible. The leaders involved in the current study anticipated that daily registration in the digital ethical reflection tool would increase staff awareness of and improve how they present their values in their language within the work unit and improve their familiarity with ethical principles.^51^ Language also represents the power instituted by the norms that are controlled by discourse within the workplace. Language represents and displays the work climate and defines staff’s moral boundaries. Inconsiderate language from colleagues can be insufferable to those who experience it^53^ and needs to be dealt with by the leader.

Engaging staff in digital ethical reflection could be potentially challenging. Leaders already have many expectations placed on them and play several demanding roles within the organization and work unit.^54^ For a leader to successfully change staff behaviour and engage them in digital ethical reflection, strategies to improve staff motivation, such as teaming up with a professional development nurse and dedicated healthcare professionals, were considered useful. Allying with a professional development nurse or staff member whom the leader trusts and can have discussions with is vital. Structures of action that empower the leader involve using other peer networks and informal alliances. Delegating power is a leadership strategy that can influence staff’s intrinsic motivation and increase engagement in digital ethical reflection.^55^ However, the primary responsibility for systematic ethics work remains with the nurse leader.

### Strengths and limitations

All participants had been leaders for several years, and many had informal and formal leadership experience. One aspect that contributes to the strength of our study is the selection of leaders from different settings within home nursing care. The decision to include leaders from two seemingly different fields was driven by our desire to gather a wide range of perspectives and experiences, thereby increasing the potential transferability of our study. Since some participants were prevented from participating in the scheduled interviews, some had fewer participants, and one interview was conducted via an online Teams meeting. We decided to proceed with the interviews as scheduled to respect the other participants’ time. Focus group interviews involving more participants could have led to more information being collected through the iterative group process. Using online Teams meetings for focus group interviews could limit participant interactions and, therefore, impact the generalizability of the findings. Furthermore, a more gender-diverse sample could have expanded the data collected.

## Conclusions

Nurse leaders in home nursing care demonstrate a willingness to embrace digital solutions for managing ethics work. Their expectations regarding the introduction of a digital tool revolve around empowering their staff, enabling the identification of ethical issues in practice, and fostering a reflective approach by keeping the pot of systematic ethics work boiling. These findings highlight the role of individual healthcare professionals and ethics reflection groups in bringing visibility to ethical considerations through the utilization of digital tools. Future studies should focus on investigating the feasibility and effectiveness of digital reflection as an ethical tool in healthcare settings.

## Note

1 Bachelor in social education (learning disability nursing).
